# Sex-Dependent Differences in Physical Exercise-Mediated Cognitive Recovery Following Middle Cerebral Artery Occlusion in Aged Rats

**DOI:** 10.3389/fnagi.2019.00261

**Published:** 2019-09-18

**Authors:** Charles H. Cohan, Mehdi Youbi, Isabel Saul, Alex A. Ruiz, Concepcion C. Furones, Pujan Patel, Edwin Perez, Ami P. Raval, Kunjan R. Dave, Weizhao Zhao, Chuanhui Dong, Tatjana Rundek, Sebastian Koch, Ralph L. Sacco, Miguel A. Perez-Pinzon

**Affiliations:** Peritz Scheinberg Cerebral Vascular Disease Research Laboratory, Department of Neurology, University of Miami Miller School of Medicine, Miami, FL, United States

**Keywords:** stroke, brain focal ischemia, reproductive senescent female, cognition, treadmill exercise, contextual fear conditioning

## Abstract

Stroke remains a leading cause of death and disability in the United States. No current treatments exist to promote cognitive recovery in survivors of stroke. A previous study from our laboratory determined that an acute bout of forced treadmill exercise was able to promote cognitive recovery in 3 month old male rats after middle cerebral artery occlusion (MCAo). In this study, we tested the hypothesis that 6 days of intense acute bout of forced treadmill exercise (physical exercise – PE) promotes cognitive recovery in 11–14 month old male rats. We determined that PE was able to ameliorate cognitive deficits as determined by contextual fear conditioning. Additionally, we also tested the hypothesis that PE promotes cognitive recovery in 11–13 month old reproductive senescent female rats. In contrast to males, the same intensity of exercise that decrease cognitive deficits in males was not able to promote cognitive recovery in female rats. Additionally, we determined that exercise did not lessen infarct volume in both male and female rats. There are many factors that contribute to higher stroke mortality and morbidities in women and thus, future studies will investigate the effects of PE in aged female rats to identify sex differences.

## Introduction

Stroke is the 5th leading cause of death in the United States and a leading cause of disability. Those that survive strokes are often left with both motor and cognitive impairments. Currently, there is no accepted neurological treatments to promote cognitive recovery in clinical practice. One potential strategy to promote cognitive recovery after a stroke is the use of physical exercise.

Physical exercise has beneficial effects in improving cognition in children (Davis et al., 2011) and the elderly (Blanchet et al., 2018; Kennedy et al., 2018) after traumatic brain injury (Griesbach et al., 2004) and post stroke (Marzolini et al., 2013). The optimal amount of exercise or exercise type that promotes recovery is not well defined. A previous study from our laboratory indicated that an acute bout of forced treadmill exercise (30 min a day for 6 days at a speed of 10–12 m/min) promoted cognitive recovery following both cardiac arrest and stroke in 3 month old male rats (Stradecki-Cohan et al., 2017). However, stroke is an injury that commonly occurs in the elderly. This is why the STAIR criteria emphasize the importance of testing pre-clinical therapies in aged animals and also to determine sex differences (Albers et al., 2011). In this study, we aimed to investigate the ability of forced treadmill exercise to promote cognitive recovery in 11–14 month old male and 11–13 months old female rats. Furthermore, we investigated the modifying effects of exercise on post stroke infarct volume and on sex dependent differences in survival.

## Materials and Methods

### Animal Care

All experiments were performed in accordance with the Guide for Care and Use of Laboratory Animals and approved by the University of Miami Institutional Animal Care and Use Committee. Retired breeder Sprague – Dawley rats of both sexes were purchased from Charles River and were housed within the division of veterinary resources facility for the entire length of the experiment. Animals were allowed access to food and water *ad libitum*. Prior to exposure to any experimental paradigm, animals were handled by researchers for a 3 day period in order to acclimate them to human touch to reduce stress and discomfort throughout the experimental procedures. All animals were fasted overnight prior to surgery. The age of retired breeder male rats was 11–14 months at the time of MCAO.

### Female Reproductive Senescence Determination

The estrous cycles of retired breeder rats were monitored for ∼20 days prior to experimentation by daily examination of vaginal smears (de Rivero Vaccari et al., 2016). Retired breeder rats that remained in constant diestrous were considered reproductively senescent (RS) (Selvamani and Sohrabji, 2010; de Rivero Vaccari et al., 2016). The age of RS rats was 11–13 months at the time of MCAO.

### Transient Middle Cerebral Artery Occlusion (MCAO)

Age matched male and female rats were exposed to MCAO. MCAO was achieved with intraluminal suture blockage of the middle cerebral artery for 90 min as described in previous publications (Belayev et al., 1996; Lin et al., 2014). Physiological parameters were maintained within normal limits through the surgery.

### Forced Treadmill Exercise

Three days prior to undergoing MCAO surgery, rats were exposed to the treadmill for a 3 day period, in order to acclimate themselves and reduce stress in later behavioral experiments. Three days following MCAO animals began daily exercise training for 6 days (Stradecki-Cohan et al., 2017). All animals were placed on the treadmill for a 2 min warm up at a speed of 5 m/min. After the warmup period rats ran on the treadmill for a 30 min period. The exercise speeds for each group were as follows; no exercise (0 m/min), mild exercise (6 m/min), moderate exercise (9 m/min), and intense exercise (12 m/min). In our previous study, 3 month old male rats were able to run at a speed of 18 m/min for 30 min (Stradecki-Cohan et al., 2017). However, 12 month old rats were unable to run at this speed after MCAO. Therefore, we reduced the intense exercise group to 12 m/min. For female rats, only intense exercise (12 m/min) paradigm and a no exercise control group were tested. An electric shock grid (0.2 mA) was active at the back of the treadmill so that if the animal stopped running during the experiment a light electric shock was delivered to the animal as motivation to continue running. No animals were excluded for their inability to run on the treadmill in this experiment. We excluded animals that suffered respiratory dysfunction, surgery complications or died during the course of the study.

### Contextual Fear Conditioning

Twenty days following MCAO injury, rats were subjected to contextual fear condition test (Zelikowsky et al., 2012; Cohan et al., 2015; Stradecki-Cohan et al., 2017). Briefly, rats were brought into the fear conditioning room 20 min prior to testing. Animals were then placed into the operant conditioning chamber (Colbourne Instruments, United States). Animals were allowed to explore the chamber for 340 s and then animals received a 2 s 1.5 mA shock. Animals were then removed from the chamber after 30 s. The following day, animals were again returned to the same room 20 min prior to testing. Rats were then placed in the fear conditioning chamber for an 8 min period. The amount of time spent freezing during an 8 min period was measured using FreezeFrame software.

### Infarct Volume Measurement

Three weeks following MCAO, animals were perfused with saline and then with a formaldehyde, acetic acid, and methanol solution (FAM; 10:10:80). Brains were then embedded with paraffin and 10 μm thick sections were obtained. Infarct volume measurements were adapted from previous publications (Belayev et al., 2005). Briefly, the same nine coronal sections were selected for every animal (Bregma levels 5.2, 2.7, 1.2, −0.3, −1.3, −1.8, −3.8, −5, and −7.3). Infarcted area was then measured on each section using MCID software. Infarct volume was then determined similar to previously used methods (Belayev et al., 2005).

### Statistical Analysis

All figures are displayed as means with standard error of the mean (SEM) error bars. We used the Student’s *t*-test for comparisons of two groups, as well as Chi square test, or One-way ANOVA measurements with a Bonferroni *post hoc* test as indicated within the text. We used a Fischer exact test to determine the differences in infarct volumes between the specific brain regions.

## Results

### Physiological Parameters Consistent for Aged Male and Female Rats Except Body Weight

In order to determine that the severity of the injury given is uniform between animals, a number of physiological parameters were measured for both aged male (Table 1) and aged female rats (Table 2). Body weight, cranial temperature, rectal temperature, blood pH, pCO_2_ pO_2_, MABP, and blood glucose were measured before and after MCAO. We attempted to maintain physiological parameters in normal range throughout the experiment (Tables 1,2).

**TABLE 1:** MCAO physiological parameters for male rats belonging to specific exercise and no exercise groups.

**Group**	**Variable**	**Before**	**After MCAO**
		**MCAO**	
Sham (*n* = 10)	Body weight (g)	630 ± 35	
	Cranial temp (°C)	36.4 ± 0.17	36.8 ± 0.12
	Rectal temp (°C)	36.5 ± 0.28	37.0 ± 0.03
	pH	7.37 ± 0.07	7.40 ± 0.06
	pCO_2_ (mmHg)	38.9 ± 6.56	36.4 ± 4.69
	pO_2_ (mmHg)	119 ± 18	144 ± 36
	MABP (mmHg)	122 ± 9	111 ± 11
	Blood glucose (mg/dl)	142 ± 26	155 ± 50
MCAO + No exercise (*n* = 11)	Body weight (g)	634 ± 51	
	Cranial temp (°C)	36.4 ± 0.29	36.6 ± 0.35
	Rectal temp (°C)	36.4 ± 0.25	36.8 ± 0.29
	pH	7.40 ± 0.04	7.38 ± 0.05
	pCO_2_ (mmHg)	38.8 ± 3.13	39.0 ± 5.83
	pO_2_ (mmHg)	127 ± 31	131 ± 17
	MABP (mmHg)	117 ± 7	105 ± 10
	Blood glucose (mg/dl)	167 ± 36	164 ± 34
MCAO + Mild exercise (*n* = 10)	Body weight (g)	617 ± 68	
	Cranial temp (°C)	36.3 ± 0.16	36.8 ± 0.60
	Rectal temp (°C)	36.5 ± 0.25	37.0 ± 0.43
	pH	7.40 ± 0.07	7.43 ± 0.05
	pCO_2_ (mmHg)	37.5 ± 3.65	35.6 ± 5.53
	pO_2_ (mmHg)	120 ± 35	132 ± 23
	MABP (mmHg)	117 ± 11	116 ± 15
	Blood glucose (mg/dl)	138 ± 25^∗^	144 ± 40
MCAO + Moderate exercise (*n* = 11)	Body weight (g)	632 ± 65	
	Cranial temp (°C)	36.2 ± 0.19	36.7 ± 0.55
	Rectal temp (°C)	36.5 ± 0.17	37.0 ± 0.50
	pH	7.41 ± 0.04	7.39 ± 0.04
	pCO_2_ (mmHg)	40.2 ± 3.69	39.4 ± 4.04
	pO_2_ (mmHg)	121 ± 35	132 ± 31
	MABP (mmHg)	120 ± 7	112 ± 18
	Blood glucose (mg/dl)	145 ± 41	170 ± 48
MCAO + Intense exercise (*n* = 11)	Body weight (g)	627 ± 66	
	Cranial temp (°C)	36.3 ± 0.25	36.6 ± 0.24^#^
	Rectal temp (°C)	36.3 ± 0.19	36.7 ± 0.16^#,#^
	pH	7.40 ± 0.04	7.39 ± 0.04
	pCO_2_ (mmHg)	40.9 ± 3.93	37.4 ± 3.07
	pO_2_ (mmHg)	136 ± 26	129 ± 24
	MABP (mmHg)	115 ± 15	110 ± 14
	Blood glucose (mg/dl)	141 ± 37	141 ± 41

*^∗^*p* < 0.05 vs. no exercise group. #*p* < 0.05 vs. sham group. #,#*p* < 0.001 vs. sham group.*

**TABLE 2:** MCAO physiological parameters for female rats belonging to exercise and no exercise groups.

**Group**	**Variable**	**Before MCAO**	**After MCAO**
Sham (*n* = 10)	Body weight (g)	353.4 ± 31	
	Cranial temp (°C)	36 ± 0.49	36.77 ± 0.46
	Rectal temp (°C)	36 ± 0.24	36 ± 0.39
	pH	7.4 ± 0.03	7.42 ± 0.04
	pCO_2_ (mmHg)	41.2 ± 2.7	36.6 ± 1.35
	pO_2_ (mmHg)	152.5 ± 30.7	136.25 ± 18.3
	MABP (mmHg)	142.7 ± 8.6	127 ± 17
	Blood glucose (mg/dl)	172.56 ± 38	164.12 ± 56
MCAO + No exercise (*n* = 10)	Body weight (g)	363.4 ± 43	
	Cranial temp (°C)	36 ± 0.46	36.8 ± 0.5
	Rectal temp (°C)	35.5 ± 1.25	36 ± 0.43
	pH	7.43 ± 0.07	7.37 ± 0.04
	pCO_2_ (mmHg)	38.18 ± 2.4	42.52 ± 1.6
	pO_2_ (mmHg)	125.41 ± 32.04	120.8 ± 16.76
	MABP (mmHg)	144 ± 18	134 ± 30
	Blood glucose (mg/dl)	172.8 ± 31	196.1 ± 23
MCAO + Exercise (*n* = 11)	Body weight (g)	365.1 ± 44	
	Cranial temp (°C)	36 ± 0.23	36.8 ± 0.24
	Rectal temp (°C)	36 ± 0.1	36 ± 0.55
	pH	7.42 ± 0.03	7.38 ± 0.02
	pCO_2_ (mmHg)	37.8 ± 3.83	41.33 ± 2.68
	pO_2_ (mmHg)	111.95 ± 37	104.8 ± 11.03
	MABP (mmHg)	139.47 ± 9.9	136.0 ± 12.7
	Blood glucose (mg/dl)	175 ± 20	198.75 ± 10.26

**FIGURE 1:**
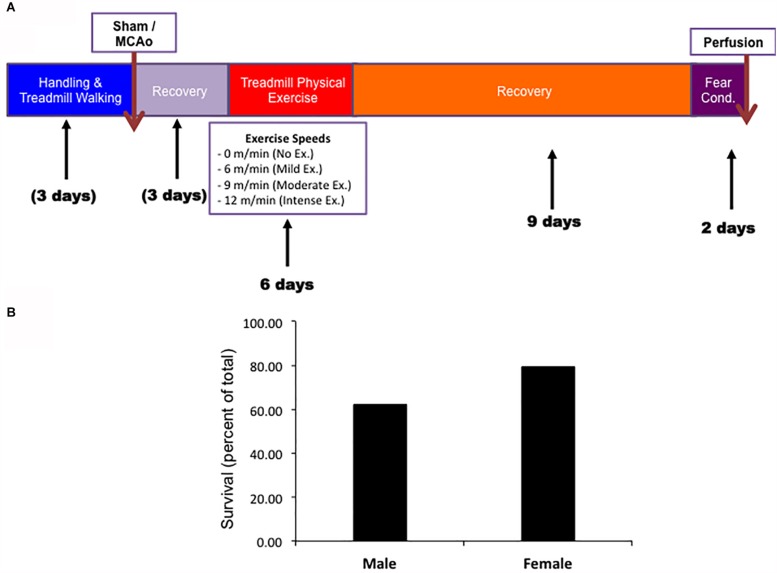
**(A)** Timeline of experimental procedures for both male and female experimental groups. **(B)** Survival rates for both male and female animals.

Both males and females underwent the same experimental paradigm. Survival rates throughout the paradigm were observed for aged males and female animals prior to exercise (Figure 1A). Male and female animals demonstrated a three week survival rate of 62% (54/89) and 79% (31/39), respectively. All mortality except for two animals (one male and one female) occurred within 4 days of MCAO surgery (prior to exercise treatment). The survival rate was higher in aged females than in aged males (*P* < 0.05, Chi Square, *n* = 134, 89 male, 39 female). No animal in the sham MCAO surgery group died (male *n* = 10 and female = 10).

### Forced Treadmill Exercise Improves Cognitive Recovery in Aged Male but Not in Aged Female Rats

To determine the importance of an acute bout of physical exercise on long term cognitive outcomes in aged male and female animals, we employed a previously used paradigm developed in our laboratory (Stradecki-Cohan et al., 2017). This paradigm uses an acute bout of forced treadmill training, followed by assessing cognitive recovery and infarct volume at around 3 weeks post-MCAO (Figure 1A). In order to determine cognitive recovery a contextual fear conditioning task measuring freezing response was determined at the end of the 3 week survival period (Figure 1B). Previous protocols found that contextual fear conditioning was a sensitive measure of determining cognitive recovery in rats that had undergone cerebral ischemia (Cohan et al., 2015; Stradecki-Cohan et al., 2017).

**FIGURE 2:**
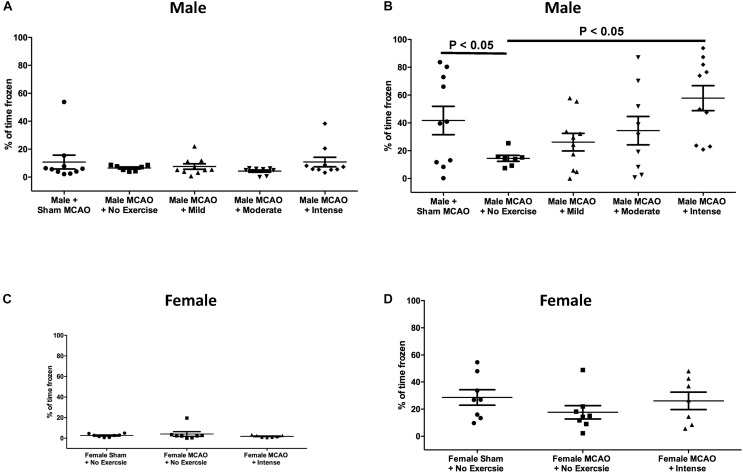
Contextual fear conditioning as a measure of spatial memory performance 3 weeks following MCAO injury in aged male and female rats. **(A)** Day 1 as a percent of time spent frozen prior to exposure of shock stimulus for aged male rats. There were no significant differences between groups (One way ANOVA, Bonferroni *post hoc*). **(B)** Day 2, 24 h following exposure to shock in the same context for aged males. Animals that underwent intense exercise froze significantly more than animals that received no exercise (*P* < 0.05, One way ANOVA, Bonferroni *post hoc* test). **(C)** Day 1 as a percent of time spent frozen prior to exposure of shock stimulus for aged female rats (Students *t*-test). **(D)** Aged female rats at the same time point with or without intense exercise. There was no observed benefit for aged female animals.

In aged rat males, day 1 prior to the shock, freezing response to the context was measured in order to determine whether there was any baseline mobility or anxiety toward the context for aged males (Figure 2A). Rats that underwent sham surgery (*n* = 10) froze 10.7 ± 4.9% of the time, whereas animals that underwent MCAO but had no exercise (*n* = 7) froze 6.5 ± 0.8% of the time. Mild exercise animals (*n* = 10), moderate exercised animals (*n* = 9), and animals that underwent intense exercise (*n* = 10) froze 7.6 ± 1.9%, 4.3 ± 0.8%, and 10.8 ± 3.4% of the time, respectively. There were no observed significant differences between any of the groups for male rats on day 1 (1 way ANOVA, Bonferroni *post hoc*). Following contextual fear conditioning paradigm, rats were then tested again 24 h later. Rats that underwent sham surgery froze 41.7 ± 10.2% of the time. Intensely exercised rats froze 57.8 ± 9.0% of the time, which was significantly higher than animals that received an MCAO injury but did not exercise, which froze 14.5 ± 2.2% of the time (*P* < 0.05, 1 way ANOVA, Bonferroni *post hoc*). There was no significant difference between the mild exercise group (froze 26.2 ± 6.3% of the time), moderate exercise group (34.4 ± 10.2% of the time), and the no exercise group (14.5 ± 2.2% of the time).

Based on these findings, we proceeded to test the hypothesis that the intense rate of exercise also improves contextual fear conditioning response in aged female rats. Rats on Day 1 of the context prior to shock exhibited minimal freezing. Female rats that underwent sham surgery froze 2.5 ± 0.57% of the time. Female rats that underwent MCAO surgery but did not exercise froze for 4.1 ± 2.3% of the time as compared with rats that received exercise post MCAO who froze 1.8 ± 0.4% of the time (*P* > 0.05, Students *T*-test). When animals were returned to the context on the second day to assess cognitive recovery, freezing results were different for female rats (Figure 2D), as compared to aged male rats. Female rats that underwent sham surgery froze 28.63 ± 5.7% of the time on second day. For female animals, the exercise intensity that promoted cognitive recovery in males showed no significant difference in freezing response (26.1 ± 6.4% of the time) compared to a no exercise MCAO control (17.7 ± 4.9% of the time). This difference indicates that forced exercise in aged males and females have different outcomes on promoting cognitive recovery following MCAO injury.

### Forced Treadmill Exercise Does Not Reduce Infarct Volume in Aged Male or Female Rats

**FIGURE 3:**
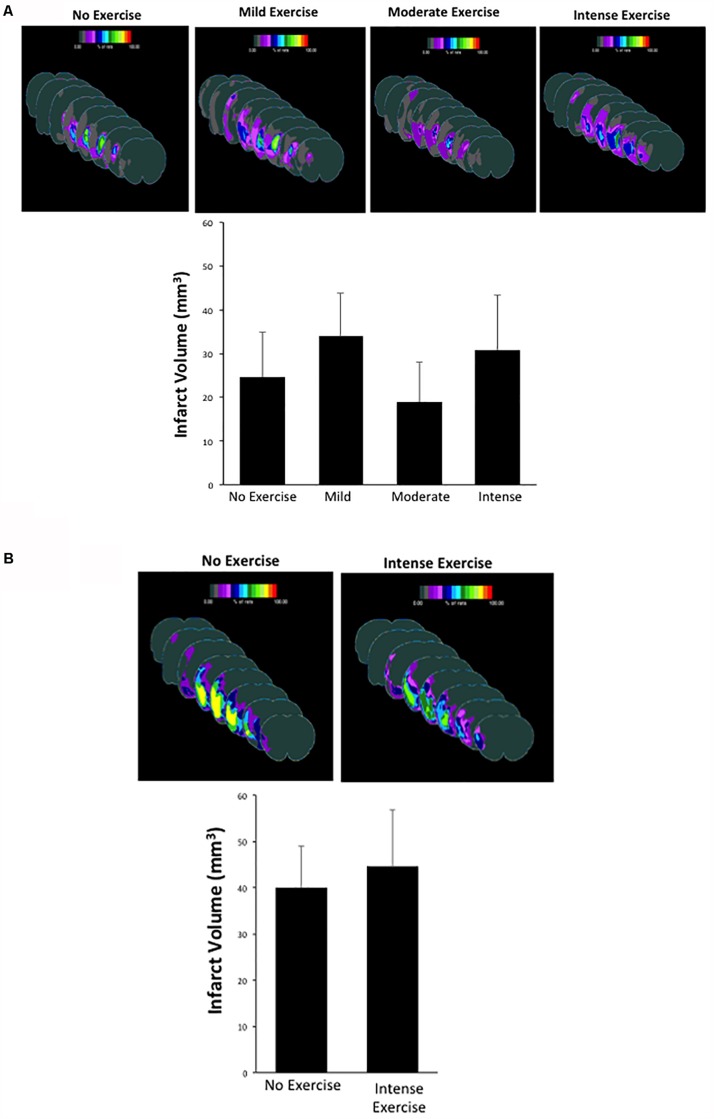
**(A,B)** Infarct volume measurements observed in male and female rats that had undergone different exercise intensities following recovery from MCAO. There were no significant differences observed between groups (one way ANOVA, Bonferroni *post hoc* test).

One potential explanation for the improved behavioral outcome is the role of exercise in promoting a reduction in the infarcted area (Dang et al., 2011). This hypothesis was based on the fact that exercise can promote increased levels of neurotrophic factors such as BDNF, which can help reduce infarct volume over time (Zhang and Pardridge, 2006). Thus, we measured the infarcted area in each group at the three-week time point after MCAO as a secondary outcome (Figure 3). Sham male and female animals showed no infarction (data not shown). For aged male rats, animals that underwent MCAO + no exercise had an infarct volume of 24.64 ± 10.31 mm^3^. This volume was not significantly different in the mild exercise group (34.03 ± 9.81 mm^3^), the moderate exercise group (18.93 ± 9.15 mm^3^), or the intense exercise group (30.77 ± 12.69 mm^3^) (*P* > 0.05, one way ANOVA, Bonferroni *post hoc*) (Figure 3A). For aged female rats, animals that did not exercise displayed an infarct volume of 39.99 ± 9.07 mm^3^, which also was not significantly different from animals that underwent intense exercise 44.68 ± 12.05 mm^3^ (Figure 3B). Exercise did not significantly increase total volume of infarct in either males or females.

### Specific Cortical and Striatal Areas Show Increased Injury in Aged Male Rats

**FIGURE 4:**
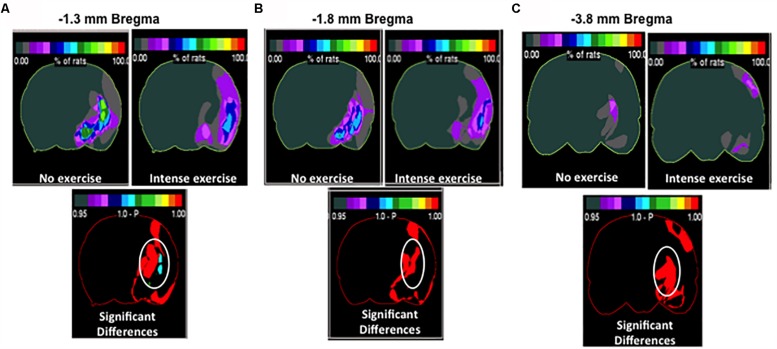
Region specific infarct volume in aged male rats. Frequency heat map representation of infarct volume for each treatment group depicting location of region specific injury. Non-circled red is an increase in injury in the intense group. **(A)** Significant decrease in injury in the intense exercise group at –1.3 mm bregma was observed. There was an observed decrease in injury in the thalamus striatal injury and basal nucleus of Meynert. Non-circled red is an increase in injury in the intense group. **(B)** Highlighting –1.8 mm bregma showing a decrease in thalamic injury **(C)** –3.8 mm bregma showing a decrease in thalamic injury. Non-circled red is an increase in injury in the intense group.

Although we determined that there was no significant difference in overall volume changes, we wanted to investigate region specific changes in infarcted region. Region specific changes may explain the observable cognitive recovery promoted by intense exercise in the aged male rat cohort (Figure 4). Interestingly, intense exercise was protective in a number of regions when compared to the no exercise group (Figures 4B,C). There were significant reductions in infarct volume found within the thalamus, and the striatum (Figures 4B,C), the globus pallidus, and within the basal nucleus of Meynert (Figures 4B,C) in intense exercise group compared to no exercise group. These changes highlight specific areas of focus that may explain some of observed cognitive performance on the contextual fear conditioning test 3 weeks after injury.

## Discussion

Stroke affects mostly aged individuals (Benjamin et al., 2018). Aging may worsen outcomes by increasing the severity of the deficits (Rosen et al., 2005). Preclinical work investigating the best outcomes after stroke in aged individuals have determined that many therapies that are effective in young animals are less effective in aged animals. Thus, the main focus of the paper is to determine whether forced treadmill exercise ameliorates cognitive impairment in aged animals of both the sexes after focal cerebral ischemia.

In our previous study, we showed that after MCAO, young male rats (3 month old) subjected to moderate exercise (10 m/min) for 6 days (as in the current study) had a significant enhancement in duration of contextual freezing compared to non-exercised rats (Stradecki-Cohan et al., 2017) indicating improved cognitive encoding/recall. In that study, we observed that moderate exercise (10 m/min) improved contextual fear conditioning response 21 days after MCAO. However, we also saw trends for improvement at the mild (6 m/min) and intense (18 m/min) exercise. As we began our studies with the aged male rats, we noticed that these rats were unable to run the treadmill at 18 m/min and that 12 m/min was the highest intensity we could use consistently in this age cohort. This level of exercise intensity was sufficient to improve recall in the contextual fear conditioning paradigm. Lower exercise intensities however, were unable to ameliorate contextual recall deficits. For example, in our previous study, 10 m/min PE intensity in young rats was able to ameliorate contextual recall deficits, but in 12 month old rats, 9 m/min had no effect. These results further stress the importance of testing stroke treatments in aged animals that more closely resemble the aged human population that suffers strokes. As we continue our studies into even older cohorts of animals, we will probably encounter additional challenges.

A key finding from our study was that the intense exercise paradigm that improved post-stroke cognition in aged male rats was not effective in improving cognitive outcomes in aged matched RS female rats. The cause of reproductive senescence in female rodents is the depletion of ovarian oocytes reserve leading to the inability of ovaries to produce hormones, progesterone, and estrogen. Especially, estrogen is crucial for neurogenesis (Galea et al., 2006), spinal plasticity (Barha and Galea, 2010; Spencer-Segal et al., 2012), long-term potentiation (Liu et al., 2008), and cognition (Barha et al., 2010). In contrast to females, in male rats, reproductive aging is not well defined, and occurs much later in lifespan resulting in decreased fertility. Since the current study used RS female and age matched male rats, the effects of estrogen on cognition might be weaned off while those of testosterone (Bimonte-Nelson et al., 2003; Jian-xin et al., 2015) may persist and reflect on improved post-stroke cognition in exercised male rats. In middle-aged rats, similar to men, exercise training increases testosterone, sex-hormone binding globulin, and endothelial nitric oxide (e-NOS) (Zmuda et al., 1996; Seo et al., 2018). Therefore, the observed improvement in post-stroke cognition in exercised male rats could be due to enhanced cerebral blood flow and testosterone mediated effects. A study testing the effects of exercise on skeletal muscles, demonstrated that exercise training improves mitochondrial function oxidative capacities in both male and female rats, but it is more pronounced in males (Farhat et al., 2017). In the case of female rats, the effectiveness of regular and moderate intensity physical exercise is age-dependent (Marosi et al., 2012) and RS female may require extended period of physical activity before the benefits on memory become apparent (Sager et al., 2018), which indicates that it is important to consider titration of exercise for female animals.

Previous studies tested a number of different exercise paradigms to promote functional recovery. In a different paradigm than the one presented in our study, forced treadmill exercise paradigms administered prior to MCAO showed some improvement in the ability to protect against injury (Hayes et al., 2008). In another paradigm, voluntary wheel running experiments promoted some benefit post stroke, showing an improvement in both motor outcomes, and the molecular profile of peri-infarct tissue reduction (Mizutani et al., 2011). Forced physical exercise, as the paradigm we used in our study, has been previously demonstrated to promote beneficial behavioral and histological outcomes after stroke. An earlier study using young rats reported that forced treadmill exercise of 20 and 30 m/min day for 14 consecutive days post-MCAO was able to lower infarct volume as well as improve motor function when evaluated on day 14 post-reperfusion (Chang et al., 2011). This study did not test for cognitive improvement. Another study in young male rats, evaluated the effect of forced physical training (rota-rod at a speed of 12 m/min for 40 min daily for 14 consecutive days) post-MCAO on functional recovery. Significant improvement on modified neurological severity scores (mNSS) was observed 17 days post-reperfusion (Lee et al., 2008). However, this study did not observe a reduction in infarct volume in the rats that were exercised. In agreement with this latter study, our studies and some others evaluating the effect of post-stoke physical exercise on infarct volume in young male rats did not observe any reduction in infarct volume (Lee et al., 2008; Stradecki-Cohan et al., 2017). This suggests that other mechanisms are at play to improve cognitive recovery.

In addition to the putative beneficial effects of sex hormones described above, there are multiple other mechanisms that may be activated following exercise post-stroke. For example, an earlier study observed that forced physical exercise using rota-rod increased neurogenesis in subgranular zone of the dentate gyrus (Lee et al., 2008). Another study evaluating the effect of voluntary exercise post-stroke observed upregulation of proteins involved in neurogenesis (Mizutani et al., 2011). Thus, these studies suggest that enhanced neurogenesis in subgranular zone may be, in part, responsible for observed enhanced cognitive performance in aged male rats subjected to post-stroke exercise in our studies.

Increased angiogenesis induced by post-ischemic exercise is another potential mechanism. Ding et al. (2004) observed that a daily 30 min (15 m/min) treadmill exercise increased levels of angiopoietin, mRNA levels of four VEGF isoforms, and microvessels density in both cortex and striatum. It is also possible that exercise-induced improvement in cognitive function may also be due to increased post-ischemic angiogenesis. As mention in the paragraph above, one of the mechanisms by which treadmill exercise confers protection against ischemic damage is via potential angiogenesis (Tang et al., 2018; Chen et al., 2019). An earlier study reported that in rodent brain, thalamic areas have higher capillary density compared to other brain areas (Xiong et al., 2017). It is plausible that more vascularized brain areas may thus have more beneficial impact of treadmill exercise compared to poorly vascularized brain area. More detailed studies are required to further confirm this hypothesis.

Although there were no differences in overall infarct volume between the different exercise groups, there were differences between specific areas in male rats. We observed significant reductions in infarct region found within the thalamus, and the cholinergic basal nucleus of Meynert in the intense exercise group as compared to no exercise group (Figure 4). In support of the current findings, a prior study observed functional disturbances and disruption of the cholinergic pathway between the frontal cortex and the basal nucleus of Meynert after middle cerebral artery occlusion in rats (Kataoka et al., 1991). Previous work has also indicated that injury to the thalamus can disrupt cognitive function (Savage et al., 2011). Injury to the basal nucleus of Meynert may play a role in mediating spatial memory function (Tian et al., 2004) and it has been proposed that damage to this nucleus is involved in the pathological mechanism of Alzheimer’s disease (Grothe et al., 2012; Shu et al., 2019). Furthermore, in a rat model of Alzheimer’s disease produced by lesion to cholinergic innervation, treadmill running delayed cognitive decline and prevented memory deficit (Hosseini et al., 2013). An increase in cortical cerebral blood flow via the activation of cholinergic neurons originating in the basal nucleus of Meynert can protect the ischemia-induced delayed death of cortical neurons by preventing a blood flow decrease in widespread cortices (Hotta et al., 2002). Therefore, it is possible that specific cell types within these regions are better protected from long term cell death occurring within the weeks following stroke. Further studies are warranted in order to determine if these regions are critical toward exercise dependent cognitive recovery.

## Summary

In this publication, we investigated the effects of aging and sex differences on different paradigms of forced treadmill exercise and their ability to modify cognitive recovery after stroke. We found that intense exercise was necessary in order to promote cognitive recovery in male animals (Figure 2). However, the same exercise intensity did not promote cognitive recovery in female animals (Figure 2). Future studies are being design to test what is the best PE intensity in young female rats. Neither of these paradigms decreased the total volume of the infarct (Figures 3, 4). The efficacy of PE in lowering post-stroke cognitive impairment remains to be establish in animal models of stroke comorbidities. Overall, this paper elucidates an exercise paradigm that could be used to promote recovery after stroke in aged male animals, however, future studies need to directly investigate specific exercise types and regimens as well as new strategies to promote the same recovery in aged female animals.

## Data Availability Statement

The datasets generated for this study are available on request to the corresponding author.

## Ethics Statement

The animal study was reviewed and approved by the Care and Use of Laboratory Animals and approved by the University of Miami Institutional Animal Care and Use Committee.

## Author Contributions

MP-P, KD, APR, CC, and MY conceived the scientific idea and designed the experiments. CC, MP-P, KD, and APR wrote the manuscript. RS, TR, and SK provided discussions on the project throughout and input in the writing of the manuscript. IS and CF performed the surgeries on rats to induce stroke. AAR, PP, and EP carried out the behavioral testing of rats. WZ and CD assisted with statistical analysis of images and data, respectively.

## Conflict of Interest

The authors declare that the research was conducted in the absence of any commercial or financial relationships that could be construed as a potential conflict of interest.
